# Gastrointestinal Parasites and the Neural Control of Gut Functions

**DOI:** 10.3389/fncel.2015.00452

**Published:** 2015-11-25

**Authors:** Marie C. M. Halliez, André G. Buret

**Affiliations:** ^1^Department of Biological Sciences, Inflammation Research Network, Host-Parasite Interaction NSERC-CREATE, University of CalgaryCalgary, AB, Canada; ^2^Protozooses transmises par l’alimentation, Rouen University Hospital, University of Rouen and Institute for Biomedical Research, University of Reims Champagne-ArdennesRouen and Reims, France

**Keywords:** gastrointestinal parasites, neuroregulation, intestinal functions, enteric nervous system, motility, secretion, absorption

## Abstract

Gastrointestinal motility and transport of water and electrolytes play key roles in the pathophysiology of diarrhea upon exposure to enteric parasites. These processes are actively modulated by the enteric nervous system (ENS), which includes efferent, and afferent neurons, as well as interneurons. ENS integrity is essential to the maintenance of homeostatic gut responses. A number of gastrointestinal parasites are known to cause disease by altering the ENS. The mechanisms remain incompletely understood. *Cryptosporidium parvum*, *Giardia duodenalis* (syn. *Giardia intestinalis, Giardia lamblia*), *Trypanosoma cruzi, Schistosoma* species and others alter gastrointestinal motility, absorption, or secretion at least in part via effects on the ENS. Recent findings also implicate enteric parasites such as *C. parvum* and *G. duodenalis* in the development of post-infectious complications such as irritable bowel syndrome, which further underscores their effects on the gut-brain axis. This article critically reviews recent advances and the current state of knowledge on the impact of enteric parasitism on the neural control of gut functions, and provides insights into mechanisms underlying these abnormalities.

## Introduction

The gastrointestinal tract (GI) serves three major functions, the digestion and absorption of the nutrients the body requires, a barrier excluding potentially harmful agents from the internal environment, and an immuno-hormonal organ to sample and manage external stimuli appropriately. Functional aspects including peristalsis, electrolyte and nutrient transport, and local blood flow are regulated by the intrinsic network of GI ganglia, the enteric nervous system (ENS; Furness et al., 1999). Motor neurons innervate the effectors systems of the gut including musculature, secretory glands and blood-lymphatic vasculature. Innervation of the muscle layer is organized into two ganglionated plexuses constituted by neurons and enteroglial cells: the myenteric plexus (Auberbach’s plexus), and the submucosal plexus (Meissner’s plexus; Furness, 2012; Furness et al., 2014). Both plexuses are embedded in the wall of the digestive tract, and consist of about 20 distinct subtypes of neurons. Those neurons can be classified as intrinsic primary afferent neurons (IPANs) that monitor the state of the lumen and gut wall, interconnecting neurons and motor neurons that target the muscle layers (Laranjeira and Pachnis, 2009). The ENS interacts with the sympathetic (*via* prevertebral ganglia) and parasympathetic (*via* the vagus nerve) neurons but constitutes an independent part of the autonomic nervous system (ANS).

The relatively small and star-shaped enteric glial cells can be identified by the presence of specific proteins such as the glial fibrillary acidic protein (GFAP), vimentin, glutamine synthetase and S100 (Gershon and Rothman, 1991). They express receptors for determined cytokines such as interleukin (IL)-1β, IL-6, tumor necrosis factor (TNF)-α and neuropeptides such as neurokinin A and substance P (SP) after activation (Gulbransen and Sharkey, 2012; Rühl, 2005). Due to these characteristics, they act together in the neuroimmune axis established in the intestinal wall, and are therefore able to modulate motility functions and GI secretions. The enteric glial cells appear to be necessary for maintenance of the structural and functional integrity of the ENS and the mucosal barrier, and are important for maintenance of gut homeostasis (by the secreting neurotrophins and cytokines) and also for neuronal interaction (Sharkey et al., 2004; von Boyen et al., 2004; Rühl, 2005; Barrenschee et al., 2013; Gougeon et al., 2013).

In the GI tract, a variety of neurotransmitters, neuroregulators and hormones play different roles in regulating the ENS and GI functions. Acetylcholine (ACh) acts through muscarinic receptors to directly affect intestinal smooth muscle contractility (Brookes and Costa, 2006; Wood, 2006). Substance P (SP), neurokinin A and neurokinin B are potent tachykinin neuromodulators and the action of SP in excitatory nonadrenergic-noncholinergic (NANC) neurotransmission is well established in the human ENS and plays an important role in nociception (Llewellyn-Smith et al., 1984). The neuroregulator Vasoactive Intestinal Peptide (VIP), which induces vasodilatation and modulates mucin release and goblet cell proliferation, participates in intestinal smooth muscle relaxation (Wood et al., 1999), stimulates intestinal secretion (Cooke, 1994; Cooke et al., 1995), and modulates immune effectors cell functions (Ottaway, 1996; Fiocchi, 1997). Cholecystokinin (CCK) is a major mediator of gastrointestinal feedback to the central nervous system (CNS) through vagal afferents (Collins, 1996; Buhner and Schemann, 2012; Schemann and Camilleri, 2013). The release of substances such as histamine, serotonin (5-hydroxytryptamine, 5-HT), and mast cell tryptase by mast cells modulates the function of a variety of intestinal cells, including nerve cells, enterocytes, as well as smooth muscle cells. Evidence has been accumulating over the years that fluid secretion in the small intestine is often evoked via stimulation of the ENS (Schemann and Camilleri, 2013). The role of the ENS in the intestinal secretory response to 5-HT and prostaglandins further supports that nerves play a key role in secretory states accompanying an inflammatory response. Somatostatin (SST), another key mast cell product, that besides regulating growth hormone secretion, inhibits secretion of many other compounds such as insulin, glucagon, gastrin, growth factors, cytokines, endocrine and exocrine secretion and as a result, regulates the activity of a broad variety of physiological processes (Reichlin, 1983a,b). SST was shown to induce both a stimulatory and inhibitory effect on cholinergic neurons of the guinea-pig ileum (Takeda et al., 1989). The pre-synaptic modulation of neurotransmitter release is an important mechanism that directly modulates intestinal motility in homeostasis. However, in pathological conditions, inflammatory mediators such as histamine may act on neuronal receptors and disrupt the normal modulation of enteric cholinergic nerve activity. These neuronal signals are conveyed from nerve endings located at different levels of the digestive wall, underlying the importance of mucosal integrity for normal transmission of afferents signals. Moreover the close apposition between vagal fibers and mast cells provides the anatomical basis for a direct neuronal communication between intestinal mast cells and the CNS (Williams et al., 1997; Buhner and Schemann, 2012; Forsythe and Bienenstock, 2012). Overwhelming evidence highlights the neuroimmune interaction between mast cells and the ENS (Collins, 1996; Buhner and Schemann, 2012; Schemann and Camilleri, 2013). This interactions between inflammatory mediators and enteric cholinergic nerves may contribute to motility disturbances observed in chronic inflammatory intestinal diseases (Hasler, 2006; Sarna and Shi, 2006).

Interactions between the endocrine, nervous and immune system create a network where cytokines, hormones and neuropeptides communicate to assist the host in maintaining homeostasis (ten Bokum et al., 2000). Parasitic infections have been found to alter the ENS via three main mechanisms: (i) modification in nerves distributions; (ii) alterations of neurochemicals levels; and (iii) altered neuronal functions. *Cryptosporidium parvum*, *Giardia duodenalis*, *Entamoeba histolytica*, *Nippostrongylus brasiliensis*, *Trichinella spiralis*, *Hymenolepis diminuta*, *Schistosoma mansoni*, *Trypanosoma cruzi* and *Toxoplasma gondii* represent prime examples of parasites that alter gastrointestinal motility, absorption, and/or secretion at least in part via effects on the ENS. This review provides a critical insight into the effects of GI parasites on the ENS, its consequences on the control of GI functions, and the mechanisms involved in GI alterations induced by parasites.

### Cryptosporidium

*Cryptosporidium* is an Apicomplexan protozoa that infects the GI tract and lungs of mammals including humans, birds, reptiles and amphibians (Fayer, 2010; Leitch and He, 2012). *Cryptosporidium hominis* and *C. parvum* are the two species that infect both immunocompetent and immunocompromised humans. Although *C. hominis* seems to be limited to humans, *C. parvum* can infect humans and a wide range of hosts including livestock (Fayer, 2010). Infections are usually attributed to drinking water, but oocysts, the infective forms, have also been recovered from contaminated food (MacKenzie et al., 1995; Laberge et al., 1996). *Cryptosporidium* most commonly infects the small intestine of immunocompetent hosts, but, gastric, hepatobiliary, pancreatic and pulmonary infections may also occur in immunodeficient or immunosuppressed hosts (Blanshard et al., 1992; Leitch and He, 2012; Zu et al., 1992). *Cryptosporidium* infection is commonly associated with villus atrophy, crypt hyperplasia, infiltration of the lamina propria, chloride (Cl^−^) hypersecretion, glucose malabsorption and a reduced barrier function (Argenzio et al., 1990; Kapel et al., 1997; Klein et al., 2008; Leitch and He, 2012).

#### *Cryptosporidium* Alters Ion Transport in ENS-Dependent and Independent Manner

Cryptosporidiosis induces neutrophil influx with marked increases in prostaglandin PGE_2_ and prostacyclin PGI_2_ release in the intestinal mucosa (Argenzio et al., 1993, 1996; Laurent et al., 1998). Prostaglandins (PG) appear to be responsible for much of the altered NaCl transport in cryptosporidiosis. While PGE_2_ directly stimulates enterocyte secretion and alters NaCl transport; its effect was shown to be independent of the ENS as it neither stimulates the ENS nor is released from ENS-stimulated cells; indeed the nerve conduction blocker, tetradotoxin (TTX), had no effect on the PGE_2_-stimulated tissue (Argenzio et al., 1993, 1996; Laurent et al., 1998). In contrast, PGI_2_ which was also elevated in infected piglet ileum and its analog, carbacyclin, reproduced the transport alterations of the infected tissue; indomethacin (a PG synthesis inhibitor) and TTX eliminated the differences in ion transport and conductance (Argenzio et al., 1993, 1996; Laurent et al., 1998). In addition, the neural network underlying PGI_2_ stimulation involved both a cholinergic and VIPergic component as atropine and VIP antagonists reduced the carbacyclin response (Argenzio et al., 1996). Increased levels of SP and its NK1 receptor in *C. parvum* infected tissue are responsible for glucose malabsorption and Cl^−^ secretion (Hernandez et al., 2007). While the mechanisms remains incompletely understood, SP is known to stimulate pro-inflammatory cytokines including interferon gamma (IFN-γ), IL-1β and TNF-α, which are known to contribute to the pathophysiology of cryptosporidiosis. Peptide YY (PYY), a powerful inhibitor of intestinal secretion mediated by VIP and PGE_2_, has been shown to completely block the malabsorptive and secretory effects of PGI_2_ analogs acting through the ENS in *Cyrptosporidium*-infected tissue (Argenzio et al., 1997). PYY was also shown to inhibit the effect induced by PGI_2_ while it had no effect on PGE_2_, suggesting a selective and intermediate level of control proximal to transport mechanisms.

Taken together, these data suggest a role for the ENS in the malabsorption and hypersecretion observed in cryptosporidiosis. Although the mechanisms by which the ENS influences these alterations remains unclear, neuropeptides seem to act as critical regulators of the pathophysiology of cryptosporidiosis.

### Giardia duodenalis

*Gardia duodenalis* (*syn. Giardia lamblia, Giardia intestinalis*) is an intestinal flagellated protozoan parasite of the upper small intestine. Very common worldwide, *Giardia* was recently included in the World Health Organization’s (WHO) Neglected Disease Initiative (Savioli et al., 2006; World Health Organization, 2004). *Giardia* is transmitted through the ingestion of cysts in contaminated food or water, or directly via the fecal/oral route. Giardiasis causes intestinal malabsorption and diarrhea in a wide variety of species including humans (Buret et al., 1992, 2015). The clinical manifestations of giardiasis range from asymptomatic, to acute or chronic diarrheal disease for reasons that remain obscure. When present, the clinical signs of infection may include diarrhea, nausea, weight loss, bloating and abdominal pain (Cotton et al., 2011; Roxström-Lindquist et al., 2006). Importantly, in giardiasis, pathophysiology occurs without invasion of the small intestinal tissues by the trophozoites, and in the absence of any overt inflammatory cell infiltration, with the exception of a modest increase in intraepithelial lymphocytes (IELs). In addition to acute symptoms, *Giardia* infections have also been associated with long term post-infectious sequelae that include ocular pathologies, arthritis, failure to thrive, stunting, growth retardation, cognitive function impairment in children, and chronic fatigue syndrome as well as functional gastrointestinal disorders (Halliez and Buret, 2013).

#### Enteric Nervous System-Dependent Parasite Elimination

The neuronal isoform nitric oxide synthase 1 (NOS1) that produces nitric oxide (NO) is involved in the elimination of *Giardia* (Li et al., 2006) consistent with the knowledge that NO relaxes smooth muscle and modulates intestinal motility (Barthó et al., 1992). NO has been shown to inhibit growth, encystation and excystation of *G. duodenalis* that are essential for establishing and maintain infection in the small intestine and transmission to other potential hosts (Eckmann et al., 2000). However the parasite has evolved strategies to evade NO-mediated host defenses. Indeed, *Giardia* depletes epithelial NO production by consuming arginine, from which NO is synthesized (Eckmann et al., 2000; Pavanelli et al., 2010). Further assessment of the role of NO in neurally-mediated enteric functions during giardiasis provides a fertile ground for future research.

#### *Giardia* Alters Neurotransmitter Levels

Reduced numbers of 5-HT-containing enterochromaffin cells in the duodenal mucosa, and lower levels of plasma 5-HT, have been reported in patients with persisting abdominal symptoms following *Giardia* infection (Dizdar et al., 2010). As 5-HT plays an important role in ENS development and GI motility, the expression, regulation and role of serotonin in giardiasis should be further investigated.

In acutely and chronically infected patients as well as in murine models, Giardia also increases the numbers of duodenal mucosal CCK-containing cells as well as postprandial plasma CCK by yet unknown mechanisms (Dizdar et al., 2010; Leslie et al., 2003; Li et al., 2007). Elevated and prolonged postprandial CCK release has also been reported in IBS patients (Sjölund et al., 1996). CCK plays a pivotal role in the regulation of digestive function and particularly in intestinal motility by stimulating cholinergic and afferent vagal fibers, suggesting that CCK may stimulate neuronal afferents in giardiasis. Indeed, treatment of *Giardia*-infected mice with CCK increased muscle contraction in a ketotifen-dependent fashion (a mucosal mast cell secretory antagonist); depletion of mast cell granule contents with compound 48/80 prior to CCK treatment also completely blocked the CCK-induced muscle contraction suggesting a role for mast cells in CCK modified levels during giardiasis(Li et al., 2007). In addition, CCK-release is known to trigger mast cell degranulation, which in turn increases smooth muscle contractility (Juanola et al., 1998). CCK major effect in the GI tract is to induce gall bladder contraction and delivery of bile in the small intestine. As bile is essential for *Giardia* trophozoite growth and an important regulator of the development of cyst, CCK release seems important to the parasite life cycle and to the host response (Farthing et al., 1983; Keister, 1983). Taken together these data suggest that increased CCK release in giardiasis could be responsible for the symptoms.

Giardiasis activates chloride secretion *in vitro* and in mice infected with *Giardia*, (Cevallos et al., 1995; Gorowara et al., 1992; Resta-Lenert et al., 2000) and present with impaired sodium-coupled D-glucose absorption in the duodenum, due to the dramatic reduction in microvillous and villous surface areas (Buret et al., 1992; Troeger et al., 2007a). Findings also indicate that giardiasis increases small intestinal transit, which is known to involve the ENS in other models (Galligan and Burks, 1986; Kuwahara et al., 1987; Argenzio et al., 1990; Deselliers et al., 1997; Castex et al., 1998; Li et al., 2011). Cause-to-effect relationships between giardiasis and post-infectious intestinal hypersensitivity have been recently reported in a study using neonatal rats (Halliez et al., 2014). Consistent with clinical presentations in the human host, post-infectious visceral hypersensitivity was observed in the small intestine as well as in the rectum, an area remote from the active colonization site by the parasite. In the acute and post-infectious phase of infection, *Giardia* facilitated the translocation of commensal bacteria, and induced the expression of the proto-oncogene *c-fos* (Chen et al., 2013; Halliez et al., 2014). Some studies have shown that noxious distension of hollow viscera (i.e., colorectum, esophagus and stomach) induces specific pattern of *c-fos* expression in the rat spinal cord and some of brain nuclei (Traub et al., 1992) suggesting neuronal activation in the CNS during visceral hypersensitivity. Finally, recent observations indicate that secretory-excretory cysteine proteases from *G. duodenalis* cleave the potent pro-inflammatory chemokine CXCL-8 to dampen host inflammation (Cotton et al., 2014a,b). Whether and how these parasite proteases may affect neuroregulation in the gut has yet to be uncovered.

### Entamoeba

Amoebiasis can manifest as amoebic colitis and/or amoebic liver abscess. The causative agent of amoebiasis, *E. histolytica*, is a widespread protozoan parasite that is endemic in developing countries (Lejeune et al., 2009; Mortimer and Chadee, 2010a,b). Transmission occurs *via* ingestion of infective cysts through contaminated food or water. Once in the GI tract, upon excystation, trophozoites are released, mostly in the terminal ileum, from where parasites migrate to the colon, where they colonize the mucus layer and actively feed on the luminal contents of the gut and the commensal resident microflora. Most often, amoebic trophozoites co-exist commensally in the host without causing any intestinal pathology. In a few cases, amoebic trophozoites destroy the colonic mucosa. In these instances, after invading the mucosa and submucosa, trophozoites may enter the portal circulation and invade the liver and other organs such as the lungs and brain, which can be fatal (Lejeune et al., 2009; Mortimer and Chadee, 2010b). *E. histolytica* trophozoites degrade the mucus layer to adhere to and lyse epithelial cells and invade leukocytes (Chadee and Meerovitch, 1984; Moncada et al., 2003, 2005; Lejeune et al., 2009; Mortimer and Chadee, 2010b).

#### *Entamoeba* Cysteine-Protease Induces Neurons and Axons Degradation

*E. histolytica* alters active electrolyte transport, secretion and malabsorption (McGowan et al., 1983; Ravdin, 1989; Keller et al., 1992; Tse and Chadee, 1992; Rana et al., 2004), but little is known on the direct effect of the parasite on the ENS. *In vitro, E. histolytica* has been found to degrade neurons from the ENS in a cysteine-protease dependent fashion (Lourenssen et al., 2010). The mechanisms as well as the specific cysteine protease involved require further investigation. Findings available to date indicate that *Entamoeba histolytica* Cysteine-Protease 5 (EhCP5) may not be responsible for this effect (Lourenssen et al., 2010). Although direct damages were shown on enteric nerves by *E. histolytica* no correlation between those damages and the modification of gut functions such as secretion, absorption and electrolyte transport has been made. Further studies are required to characterize the consequences of this *E. histolytica*-induced enteric nerve damage.

### Nippostrongylus brasiliensis

*Nippostrongylus brasiliensis* is a gastrointestinal nematode with a simple life cycle that infects rodents, primarily mice and rats. *N. brasiliensis* is often used as a model system to study the immuno-pathophysiology of brain-gut interactions (Castex et al., 1998; Camberis et al., 2003; Aerssens et al., 2007; Soga et al., 2008). *N. brasiliensis* has an external, free-living life cycle stage, an extra intestinal somatic migration phase (from the skin to the lungs), and a parasitic intestinal phase. This nematode causes villus atrophy, crypt hyperplasia, mucosal mast cell hyperplasia (Stead et al., 1987), hypertrophy of the muscularis externa (Symons and Fairbairn, 1962; Castex et al., 1998), and activates both systemic and mucosal Th2 immune responses (Camberis et al., 2003). It also induces functional changes in the intestine by impairing host protein, carbohydrate and lipid metabolism (Ovington, 1987), absorptive function of the jejunal mucosa (Carter et al., 1981; Cheema and Scofield, 1984; Nolla et al., 1985), intestinal permeability (Nawa, 1979), and electrolyte transport (Masson et al., 1996). *N. brasiliensis* also affects intestinal motility (Symons et al., 1971; Farmer, 1981; Nolla et al., 1985; Crosthwaite et al., 1990; Castex et al., 1998; Gay et al., 2001; Zhao et al., 2003, 2006), alters peptidergic neurotransmission (Masson et al., 1996), changes mucosal and nerve architecture (Stead, 1992b), increases visceral chemosensitivity (Aerssens et al., 2007) and mechanosensitivity (McLean et al., 1997).

#### *N. brasiliensis*-Induced Motility Dysfunctions are ENS-Dependent

##### Neurotransmitter-dependent ENS-modifications

Early post-infectious jejunal motor impairment was associated with decreased responsiveness of the circular smooth muscle to both cholinergic stimulation and β-adrenergic inhibition triggered by the concurrent inflammatory response (Castex et al., 1998). In contrast, 8 days post-infection, contractility of the intestinal longitudinal muscle increases. A few days later, post expulsion stage, migrating myoelectric complexes profiles gradually return to normal. The distribution of *c-fos* in the CNS during infection correlates with the onset of acute inflammation and the spatial location of the inflammatory stimulus, indicative of a strong influence of inflammatory mediators on the activation of the brain-gut axis (Stead et al., 1987; Castex et al., 1998).

Increased motility induced by *N. brasiliensis* infection involves cholinergic mediation, a vagal pathway, and alteration in intestinal CCK-A and CCK-B receptors (Gay et al., 2001). The neuroimmune changes of the gut wall following the expulsion phase of *N. brasiliensis* infection are independent of intestinal mast cell degranulation (Gay et al., 2001). Following worm expulsion, *c-fos* expression increases upon CCK stimulation, establishing a link between CCK and the activation of the brain-gut axis (Gay et al., 2002). These observations are consistent with other findings which showed that CCK activation of *c-fos* occurred via capsaicin-sensitive vagal afferents and CCK-A receptors (Mönnikes et al., 1997).

##### Immune-dependent ENS modulation

*N. brasiliensis* infection in mice induces a strong IL-4 – and IL-13—associated Th2 cytokine response that causes worm expulsion through an IL-4Rα-activated, Stat6-dependent mechanism (Urban et al., 1998). IL-4/IL-13 modulate smooth muscle responses to nerve stimulation (Goldhill et al., 1997; Akiho et al., 2002). Addition of atropine, which blocks muscarinic cholinergic nerve stimulation, attenuates the contractility induced by IL-13, but not by IL-4. Moreover addition of TTX completely blocks the hypercontractility supporting the hypothesis that enteric nerves mediate the Stat6-dependent effects of IL-13 in response to ACh (Zhao et al., 2003). An effect on noncholinergic nerves is also observed as increased responses to nerve stimulation still persist in the presence of atropine. Furthermore, IL-13 enhances response to SP which could explain that the portion of enhanced response to nerve stimulation is both atropine resistant and Stat6 dependent. The IL-4-dependent contractility mechanism appears to be completely different as it does not involve increased sensitization to ACh or SP. It was shown to be dependent on the mast cells release of leukotriene D_4_ which increased contractility by enhancing the enteric nerve sensitivity to neurotransmitters (Madden et al., 2002; Zhao et al., 2003). Although both IL-4 and IL-13 have a role in *N. brasiliensis* expulsion, there are some stimulatory effects on intestinal smooth muscle that cannot be linked to either cytokine.

#### *Nippostrongylus* Infection Induces ENS Nerve Remodeling

*N. brasiliensis* induces nerve remodeling in the gut, illustrated by a significant increase in the number of axons and an enlargement of nerve profiles (Stead et al., 1991; Stead, 1992b). Mucosal nerve remodeling in rats infected with *N. brasiliensis* occurs in two phases: (1) initial degeneration of a proportion of the mucosal nerves during the acute phase of inflammation and (2) a subsequent reinnervation phase after parasite expulsion. Observations of the nerves profiles revealed a large increase in the proportion of small nerve fibers and a reduction in the frequency of large nerve between D18 and D28 PI. This shift to smaller fibers is consistent with nerve regeneration. The degeneration during the acute inflammatory phase and regeneration thereafter is coordinated with the phases of mast cell degranulation and subsequent hyperplasia suggesting an important role of mast cell products in nerve remodeling. These data show an important communication process between the immune and nervous systems during *Nippostrongylus* infection that offer a favorable ground for further evaluation.

#### Altered Fluid Transport is Due to Inflammatory Mediators Activation of the ENS

During the acute phase of *N. brasiliensis* infection, fluid transport is impaired in infected animals, while after worm expulsion fluid transport becomes significantly greater than in uninfected animals (Jodal et al., 1993). The decreased fluid transport observed during the acute phase is at least partly mediated via activation of the ENS, since administration of hexamethonium (nicotinic receptor blocker) and lidocaine (local anesthetic) restore fluid absorption. The relative roles of components such as histamine, serotonin, prostaglandins and leukotrienes released by the mast cells in the activation of the ENS during the infection require further investigation. Indeed, such inflammatory mediators have been shown to stimulate enteric nerves either directly or via the formation of prostaglandins (Hirst and Silinsky, 1975; Schulzke et al., 2010; Smith et al., 2014).

#### *Nippostrongylus* Induces Alteration in Chemo- and Mechanosensitivity

In infected mice submitted to mild stress, *N. brasiliensis* increases sensitivity to intraluminal acid, indicating alterations of chemosensitive afferents (Aerssens et al., 2007). Acid challenge resulted in increased nerve discharge. In addition, afferent nerve firing remained elevated after acid challenge. Intraluminal pressure was also substantially increased in infected animal. However no change in mesenteric afferent activity was observed suggesting a mechanism independent of extrinsic reflex activity. The mechanism of this altered response although not investigated in this study could be due to permeability changes and/ or alteration in the excitability of intrinsic neuronal reflexes. Previous findings indicate that vagal afferent pathways play an important role in gastric chemonociception, suggesting that exacerbated acid sensitivity induced by the parasite may be explained by sensitization of vagal visceral sensory neurons (Schuligoi et al., 1998; Holzer, 2003; Aerssens et al., 2007). Furthermore, infection also alters the expression of genes of the vagal pathway known to be implicated in chemosensitivity, such as acid-sensing ion channels, transient receptor potential cation channels (TRPV1 and TRPA1), ionotropic purinoreceptor ion channels, and acid-sensitive 2-pore domain potassium channels (Aerssens et al., 2007). TRPV1 is an important regulator of mechanical and chemical hyperalgesia (Miranda et al., 2007). Jejunal hypersensitivity to capsaicin is also seen in *Nippostrongylus*-infected rats, consistent with an effect on TRPV1 (Mathison and Davison, 1993). TRPA1 is expressed in visceral afferent neurons, and is known to participate in inflammatory responses and the development of hypersensitivity (Lapointe and Altier, 2011). Gene expression for gastrointestinal peptide receptors such as CCK-A receptors, neuropeptide Y receptors (NPY_2_), the neurotensin receptor 1, the somatostatin receptor 2, GABA_A_ receptors Beta 3 subunit, and the serotonin receptor 5-HT_3A_, are also altered by infection. While 5-HT_3_ antagonists are known to inhibit chemical but not mechanical pain in rats (Botella et al., 1998), the decreased expression of serotonin receptor 5-HT_3A_ observed in infected mice here could be considered as an antinociceptive adaptation. In addition, infected rats also exhibit increased sensitivity to distension where mast cell hyperplasia occurs, in a tachykinin receptor NK2-dependent manner, and independently of inflammation (McLean et al., 1997). It remains to be seen whether changes in SP and/or in tachykinin NK_2_ receptors are implicated in these effects (Masson et al., 1996; Stead, 1992b). The role of mast cells in visceral hypersensitivity due to infection also warrants further investigation (Stead et al., 1987; McLean et al., 1997; Aerssens et al., 2007).

### Trichinella spiralis

The nematode *T. spiralis* infects a variety of mammals including humans, rodents, horses, bears and pigs. *Trichinella*-infections are characterized by two phases: an enteral phase where the adult alters intestinal functions, and a parenteral phase which is associated with muscle invasion by the larval parasites. The enteral phase lasts for about 2 weeks during which the parasites live in the intestinal epithelium. Adult worms colonize the upper intestine (duodenum and jejunum) and develop quickly to produce the infective larvae that migrate to muscle tissues. Gastrointestinal symptoms are the first to appear and include nausea, vomiting, epigastric pains, diarrhea and/or constipation. *Trichinella*-infection, similar to *Nippostrongylus*-infection, induces an inflammatory response, mastocytosis, and alteration of nutrients absorption, alteration of muscle cell structure and contractility, and intestinal hypersensitivity. The infection also affects remote segments free of parasites and of inflammation like the ileum (Marzio et al., 1990; Tanović et al., 2006).

During *Trichinella* infection, changes in the smooth muscle cells are observed and include altered cellular calcium utilization (Vermillion and Collins, 1988) and suppression of Na^+^K^+^ ATPase pump activity (Muller et al., 1989). *T. spiralis* infection also results in alterations of muscle contractility and enteric neurotransmission that persist after the resolution of the mucosal inflammation and expulsion of the parasite (Alizadeh et al., 1987; Weisbrodt et al., 1994; Venkova et al., 1999; Venkova and Greenwood-van Meerveld, 2006). Several components have been shown to be involved in the altered muscle contractility observed during and after *Trichinella* infection, those include structural (muscle thickness, hypertrophy and hyperplasia), immunological (mastocytosis) and neuronal components (altered neurotransmitter release and activity).

#### *Trichinella* Infection Induces Immune-Mediated Altered Neurotransmitters Levels

During *T. spiralis*-induced inflammation SP and VIP are significantly reduced in the jejunum and in the ileum of infected ferrets (Palmer and Greenwood, 1993; Greenwood and Palmer, 1996). In contrast, in murine Trichinellosis SP was shown to be significantly increased concurrently with high myeloperoxidase levels, and was associated with impaired lymphocyte responses to exogenous SP. Elimination of the SP-increased levels was shown to spare the murine GI tract from much of the pathologies associated with *Trichinella* infection by reducing the inflammation, consistent with the role of SP as an important modulator of GI inflammation (Kataeva et al., 1994). Similar results were observed in the rat, where *Trichinella* infection was associated with a marked increase in SP immunoreactivity in the gut wall but also in the dorsal root ganglions and dorsal horn of the spine. Addition of capsaicin, steroid, or use of athymic animals depleted SP, suggesting that neuronal changes observed in *Trichinella* infected animals is rather due to the host immune response than to the parasite *per se* (Swain et al., 1992). SP depletion also prevented the cardioautonomic response to visceral distension supporting the important role of SP in this response (De Giorgio et al., 2001). The role exerted by SP is also related to modulation of the NK-1, NK-2 and NK-3 tachykinin receptors in the spinal cord. Indeed *Trichinella*-induced inflammation evoked a decrease in NK-1 immunoreactivity in the dorsal horn’s nerve fibers and neurons, leading to desensitization and reduced neural excitability (De Giorgio et al., 2001). Taken together these data underscore the key role played by SP in the neuro-inflammatory response to *Trichinella* SP.

#### Physiological Effects of Altered Neurotransmitter Levels in Intestinal Contractility

In the rat, *T. spiralis-*infection is accompanied by a reversible suppression of ACh release from the longitudinal muscle-myenteric plexus of the jejunum (Collins et al., 1989). In another study, the contractile responses to KCl and ACh were increased from day 6–23 PI, and correlated with changes in the muscle layer thickness, suggesting that the increase in induced-contractility might be a consequence of the hypertrophy and/or hyperplasia (Tanović et al., 2006). However in mast cell deficients rats, the imbalance of ACh/SP was not detected suggesting an important role for mast cell in peptidergic remodeling of the rat ileum (Leng et al., 2010). Pharmacological analysis revealed that muscle tension responses in longitudinal smooth muscle from *T. spiralis* infected rats are associated with altered 5-HT receptor function. Infection with *Trichinella* increases mucosal 5-HT availability and affects the spontaneous activity and mechanosensitivity of GI sensory nerves (Keating et al., 2008). Endogenous 5-HT release has been shown to contribute to the basal activity of jejunal afferents *via* 5-HT_3_ receptor activation as this activity was susceptible to partial blockade with granisetron (Hillsley et al., 1998). Hypersensitivity observed in the post-infectious period was in part mediated by 5-HT acting *via* 5-HT_3_ receptors as the use of granisetron significantly attenuated the total distension response at D28–36 PI (Keating et al., 2008). However, granisetron had no effect on the total distension response at D48–56 PI indicating that the hypersensitivity observed later in the infection has a mechanism not mediated by 5-HT_3_, consistent with data from previous studies (Hillsley and Grundy, 1998; Hillsley et al., 1998; Hicks et al., 2002; Coldwell et al., 2007). After reconstitution with splenic mononuclear cells, increased tension to carbachol or 5-HT was observed post-infectiously indicating that the smooth muscle function changes following *Trichinella* infection in the rat are T-lymphocyte dependent (Vermillion et al., 1991). Several studies suggest that remodeling of the neural secretomotor pathway seen after infection may lead to persistent alterations in epithelial barrier function. Infected animals have a reduced response to 5-HT suggesting an alteration in serotonin-dependent signaling. In normal conditions, serotonin-induced electrogenic responses result from a combination of neurally mediated and direct effects on epithelial cells (Hansen and Witte, 2008). TTX normalizes the response observed in infected animals suggesting that the alterations in 5-HT-elicited current changes in infected rats are likely due to an altered epithelial function (Fernández-Blanco et al., 2011). In the post-infectious stage secretory responses to mucosal mast cells are also reduced, together with the alterations in epithelial and neural responses, which may illustrate a feedback regulatory mechanism aiming to minimize a state of continuous epithelial overstimulation. In the enteroglial cells of myenteric ganglia and macrophage-like cells of *T. spiralis*-inflamed intestines, pro-inflammatory cytokines such as IL-1β, IL-6 and TNF-α are significantly elevated (Khan and Collins, 2006). Those pro-inflammatory cytokines can act directly or indirectly through host-derived intermediate molecules such as cyclooxygenase metabolites, which may alter neurotransmitter release in the ENS and significantly affect gut function (Hurst and Collins, 1993; Hurst et al., 1993; Rühl et al., 1994). IL-5 also contributes significantly to the long-term maintenance of *Trichinella*-induced hypercontractility (Vallance et al., 1999). Although the mediator responsible for the synaptic inhibition has not been identified, the excitability phenomenon is mediated by the release of histamine by mast cells (Frieling et al., 1994). Late stage infection in rats results in persistent post-infectious barrier dysfunction characterized by increased mucosal permeability and ion secretion, and is associated with low grade inflammation which resemble findings in patients with functional gastrointestinal disorders such as IBS (Fernández-Blanco et al., 2011). Whether or not some of these post-infectious phenomena may be due to gut microbiota changes induced by the parasite has yet to be seen.

### Hymenolepis

*H. diminuta* also known as the rat tapeworm is a parasite of the small intestine of rodents (mostly rats and mice), and can also accidentally infect humans where it usually remains asymptomatic. However abdominal pain, irritability, itching and eosinophilia have also been reported in infected humans. This tapeworm has no hooks to damage host tissue and is non-invasive but metabolites produced by *H. diminuta* have been shown to disrupt the action of the GI tract, and may increase salivary secretion, inhibit gastric secretion, and activate trypsin in the chyme of the duodenum (Uglem and Just, 1983).

#### *Hymenolepis diminuta* Induces Changes in Intestinal Contractility Dependent on the ENS Integrity

Infection with *H. diminuta* in the rat alters myoelectric patterns and increases smooth muscle thickness and crypt depth in the duodenum, jejunum and ileum (Dwinell et al., 1998). The mechanisms responsible for thickening of the muscularis externae and smooth muscle hypertrophy remain unclear, but may reflect altered intestinal contractility that begins 8 days after infection (Dwinell et al., 1998). Myolectric patterns in rats chronically infected with *H. diminuta* are characterized by a concurrent decrease in normal interdigestive patterns, with the appearance of non-migrating bursts of spike potential activity and a decrease in intestinal transit (Dwinell et al., 1994, 1995, 1997). Moreover, *H. diminuta*-induced myoelectric changes resemble those observed during partial obstruction, in concordance with the fact that tapeworms occupy a significant portion of the intestinal lumen. Increased intestinal contractility may lead to smooth muscle hypertrophy after the initiation of myoelectric changes observed in *H. diminuta* infected rats (Dwinell et al., 1998). Increased mucosal mast cell populations have been observed in the ileum, the region presenting the maximal increase in intestinal myoelectric activity; however this increase was observed 2 weeks after initiation of the myoelectric changes. In addition the mucosal mast cell secretory antagonist ketotifen had no effect on the myoelectric changes observed in infected animals suggesting a mechanism independent of mast cell (Dwinell et al., 1998). As discussed above, these observations are in contrast with *Nippostrongylus* and *Trichinella* infections that involve mast cell-mediated mechanisms to alter the smooth muscle contractility (Stead et al., 1987; Weisbrodt et al., 1994).

However, integrity of the ENS is necessary for the myoelectric patterns observed during *Hymenolepis*-infection (Dwinell et al., 2002). These results suggests that “sensors” must exists along the small intestine for the initiation of the circuitry of the ENS and the generation of myoelectric patterns as transected-infected animals did not present those alterations (Dwinell et al., 2002). These myoelectric alterations were shown to be essential for the worm to remain in the rat intestine suggesting that *H. diminuta* can utilize the ENS of its host to optimize its intestinal life stage. *H. diminuta* infection increases glial derived neurotrophic factor (GDNF)-containing macrophages (Starke-Buzetti and Oaks, 2008). Considering GDNF’s involvement in neuroprotection, neural restoration, survival and remodeling, smooth muscle alterations and the ENS plasticity (nerve distribution) observed in tapeworm infection, it is logical to assume a key role for GDNF in the response to parasitic neural alterations (Starke-Buzetti and Oaks, 2008).

#### Ion Transport is Affected by *H. diminuta*-Infection

*H. diminuta* infection in the rat significantly alters the function of ion channels in the active and passive transport of ions in the ileum and colon (Kosik-Bogacka et al., 2010, 2011). Histamine, secreted by mastocytes, is able to affect ion transport by stimulating the electrogenic secretion of Cl^−^ and inhibiting the electro-neutral absorption of NaCl in the rat colon (Schultheiss et al., 2006). *H. diminuta* infection in the rat increases the numbers of mast cells, suggesting that this could play a role in the alteration of ion transport. However, the ENS, and predominantly C-fibers, are known to provide protective functions against the harmful effects of parasites in the GI tract, and non adrenergic non cholinergic neurotransmitters (SP, CGRP and others) play a critical role in preserving tissue integrity and repair (Cooke, 1994). SP stimulate mast cells to secrete inflammatory mediators including histamine that affect enteric nerve and contribute to the regulation of SP-induced ion secretion (Wang et al., 1995; Walling et al., 1977). Administration of capsaicin (which activates C-fibers) has no effect on ion transport in *H. diminuta*-infected rats which could be due to an excess stimulation of C-fibers by the parasite (Kosik-Bogacka et al., 2010). As an increase in SP has been observed in rats infected with *H. diminuta* (McKay et al., 1991; McKay and Fairweather, 1997), and as capsaicin had no effect on ion transport, the ENS and particularly C-fibers seem to be involved in ion transport alterations during *H. diminuta* infection. Nevertheless, further studies are needed to determine the mechanisms involved in C-fibers-mediated ion transport alterations.

### *Schistosoma* Species

*Schistosoma* trematodes exist as four common species: *indicum, japonicum, heamatobium*, and *mansoni* (Barker and Blair, 1996; Morgan et al., 2003). Male and female worms migrate to the portal venous system and mate in the liver where they produce large number of eggs. After 7 weeks of infection, *S. mansoni* eggs reach the mesenteric venules, and reach the terminal ileum and the colon where they induce acute inflammation (Domingo and Warren, 1969; Weinstock, 1992). The inflammatory response pushes the eggs into the lumen but some eggs may remain trapped in the tissue and lead to chronic granulomatous inflammation and result in intestinal lesions (Weinstock, 1992). Individuals with gastrointestinal schistosomiasis suffer from motility-related gastrointestinal symptoms such as diarrhea, anorexia, nausea and abdominal cramps (Rocha et al., 1995).

#### *Schistosoma*-Infection Induces Granuloma Formation and Increased Mucosal Layer Thickness

In schistosomiasis, granulomas surrounding entrapped eggs are detected in the ileal and colonic mucosa, submucosa and serosal surface. Those granulomas are mainly constituted of lymphocytes, macrophages and eosinophilic granulocytes (Bogers et al., 2000). After 12 weeks of infection, a diffuse inflammation in the mucosa may also be observed, consisting predominantly of eosinophils, neutrophils, and some lymphocytes, and causes diffuse broadening of the intestinal villi (Bogers et al., 2000; Moreels et al., 2001). Granulocytes were also detected on the outer border of ganglia of the myenteric plexus, in close proximity with neuronal cell bodies and enteric glial cells (Bogers et al., 2000). Mast cells are observed in the muscularis externae where their numbers increases with the duration of the infection. This inflammatory infiltration causes transient increases in mucosal and muscularis externae thickness (Bogers et al., 2000; Moreels et al., 2001).

#### *Schistosoma*-Infection Alters Intestinal Motility Directly and Indirectly

Intestinal transit of infected animals remains unchanged for several weeks, but at 8 weeks PI, contraction induced by exogenous contractile agonists (ACh, 5-HT, SP) is significantly enhanced in infected animals, and is further increased at 12 weeks and 40 weeks of infection, supporting long lasting effects of schistosomiasis (Bogers et al., 2000; Moreels et al., 2001). Electric field stimulation (EFS)-induced contractions in infected animals are cholinergic as they can be blocked by the muscarinic receptor antagonist atropine or TTX (neuronal conductance blockers; Bogers et al., 2000; Moreels et al., 2001). However, TTX pretreatment had no effect on spontaneous activity and receptor-mediated contractility. Therefore the effect of *S. mansoni* infection on the smooth muscle function might be either indirect through the activation of neuronal nicotinic receptors and the alteration of the myenteric plexus via granuloma-induced enteric nerve destruction (Varilek et al., 1991; Moreels et al., 2001), or direct when induced via activation of smooth muscle cell receptors (De Man et al., 2001). Contractions in ileal muscle strips were also induced by histamine. However those were not affected by TTX, atropine or hexamethonium indicating that they were not neuronal or cholinergic in origin. In addition in inflamed ileum, histamine had no effect on the nerve-mediated contraction to EFS. In contrast, an adrenergic α2-receptor agonist was able to inhibit the nerve-mediated contraction to EFS in control and inflamed tissues but had no effect on carbachol-induced contraction suggesting a neuronal action. Taken together, these observations indicate that the presynaptic modulation of cholinergic nerve activity in the ileum is disturbed during chronic schistosomiasis and that this disturbance involves nicotinic and histaminic receptors but not adrenergic α2-receptors. In the chronically inflamed ileum of *S. mansoni* infected mice, nicotinic receptors are hyper-reactive, which may activate cholinergic nerves thus disturbing the normal GI motility (De Man et al., 2001). Indeed, neurotransmitters can modulate their own release by activation of auto-receptors located on nerve terminals. In chronically inflamed ileum, hexamethonium significantly inhibited the contractions to EFS without affecting the direct smooth muscle response to carbachol supporting the idea that there is a more sensitive modulation of cholinergic nerve activity by nicotinic receptors during chronic *Schistosoma*-induced inflammation (De Man et al., 2001).

#### Immune-Mediated Contractility Alterations

The presence of mast cells in the muscularis externae, in the vicinity of the myenteric plexus could suggest a role for mast cells in altered motility during schistosomiasis. Indeed, mast cell degranulation can be initiated by various neuropeptides including SP which is also secreted by eosinophilic granulocytes that are abundantly present during *Schistosoma*-infection. Eosinophils contain large amounts of neuropeptides including SP and VIP, giving them the opportunity to interact with the myenteric plexus’ neurons (Weinstock and Blum, 1990a,b; Bogers et al., 2000). Intestinal schistosomiasis was also shown to increase the number of calcitonin gene related peptide (CGRP)-immunoreactive fibers in the lamina propria (De Jonge et al., 2003a). This up-regulation coincided with an increase in mucosal mast cells in acutely and chronically infected animals. Moreover, mucosal mast cells were found closely associated with a dense mucosal CGRP-immunoreactive fiber network in chronically infected animals (De Jonge et al., 2003a). Increased synthesis of CGRP could play a role in mucosal repair after *Schistosoma* infection. Indeed, in the rat and rabbit GI tract, extrinsic sensory neurons have a protecting role in experimental colitis by the release of CGRP (Reinshagen et al., 1994, 1998).

In addition *Schistosoma*-induced granulomas have been shown to secrete IL-1β during the early phase of their formation, and to modulate gastrointestinal neuromuscular function (Collins, 1996) suggesting a role for IL-1β in the altered contractility profiles observed in schistosomiasis. Prostaglandins, important mediators of granulomas formation and GI motility (Bennett et al., 1981; Goes et al., 1994; Eberhart and Dubois, 1995), were shown to be necessary for the maintenance of contractility inherent tone in *Schistosoma*-infection, but did not influence *Schistosoma*-induced increased contractility (Moreels et al., 2001).

In mice and guinea pig ileum, histamine inhibits the neuronally-mediated contractions to cholinergic nerve stimulation and can be prevented by histamine H_1_, but not H_2_ or H_3_, antagonists (De Man et al., 2001). The role of histamine H_1_ receptors in enteric cholinergic neurotransmission during schistosomiasis requires further investigation.

#### Neurotransmitter-Dependent Contractility Alterations

ACh release from enteric cholinergic nerves is under a well-regulated presynaptic control involving specific neuronal receptors. Among these are the purinergic receptors P1 and P2 which can inhibit or enhance the release of ACh upon activation. P1 and P2 purinoreceptors are present on immune cells, and their respective natural ligands adenosine and ATP are generated at the site of inflammation, suggesting that purines may act as neuroimmune modulators (Ribeiro et al., 1996). Chronic intestinal inflammation induced by *Schistosoma*-infection impairs the purinergic control of enteric cholinergic neurotransmission in the mouse ileum (De Man et al., 2003). In the *Schistosoma*-inflamed ileum, adenosine and ATP fail to inhibit the cholinergic nerve-mediated contractions to EFS. During the inflammatory process, purines such as adenosine and ATP, released from mast cells, can modulate mast cell degranulation and can potentiate their own effect on mast cells by a positive feedback mechanism indicating the crucial role of purines in mast cell responses to inflammation (Marquardt et al., 1978, 1984; Fozard et al., 1996). The loss of neuromodulatory action of adenosine during *Schistosoma*-induced inflammation may result from a chronic exposure of enteric nerves to adenosine released during the inflammatory process, a hypothesis further supported by the fact that pretreatement of muscle strips with methyladenosine abrogated the inhibitory effect of adenosine on cholinergic nerve-mediated contraction to EFS (De Man et al., 2003). These results suggest that purinoreceptors desensitization on mast cells can occur during prolonged contact with purines (De Man et al., 2003), in keeping with previous findings that the neuromodulatory function of histamine is impaired during chronic intestinal inflammation (De Man et al., 2001).

#### Hormone-Mediated Contractility Alterations during *Schistosoma*-Infection

The neuropeptide somatostatin expressed in nerve cells and nerve fibers of the submucosal and myenteric plexuses, has been shown to play an important role during Schistosomiasis. SST-immunoreactive nerve fibers are increased in the lamina propria of intestinal villi of infected animals (De Jonge et al., 2003b). SST-immunoreactive nerve fibers are also found within the granulomas where they could modulate inflammatory cell function through the release of neurotransmitter and *vice versa* (De Jonge et al., 2003b). *Schistosomiasis* also increases SSTR2A immunoreactivity in some neurons and nerve fibers of the myenteric plexus. In addition both SST and SSTR2A were positive for a marker of cholinergic neurons, indicating that SST and SSTR2A-expressing neurons are cholinergic populations (De Jonge et al., 2003b). Moreover other studies showed that SST and SSTR2A have an inhibitory effect on hormone and mediator secretion, exocrine secretion and cell proliferation suggesting a role for SST expressing neurons in regulation of secretion (ten Bokum et al., 2000). Pharmacological experiments showed that SST had a slight inhibitory effect during the acute stage, totally lost this effect in the chronic stage of infection, while it was able to completely inhibit EFS-mediated contractions in uninfected animals (De Jonge et al., 2003b). These observations demonstrate that there is a defective presynaptic inhibition of cholinergic transmission during chronic schistosomiasis (De Jonge et al., 2003b).

### Trypanosoma cruzi

*Trypanosoma* is a unicellular parasitic flagellated protozoan that infects a variety of hosts and causes various diseases. In humans, *T. brucei* induces sleeping sickness, while *T. cruzi* causes Chagas disease, and both can be fatal. A chronic form of Chagas’ disease may develop years after the initial infection (20–30 years), and it affects the neuro-regulation of internal organs including the heart, esophagus, peripheral nervous system and colon. The final stage of the impaired gut motility in Chagas’ disease results in dilatation of the digestive tract, and often leads to megacolon and megaesophagus, accompanied by severe weight loss due to secondary achalasia. The trypanosome induces an inflammatory response, cellular tissue lesions, and fibrosis. In addition, the amastigote stage destroys intramural neurons of the ANS that are the cause of megaintestine and cardiac aneurysms. Indeed, the digestive form of Chagas’ disease denervates the myenteric plexus. The neurological involvement is different between the acute, intermediate and chronic stages of *T. cruzi* infection and will determine the severity of the sequelae in patients (Köberle, 1968; Molina et al., 1987). Changes in the myenteric plexus of the colon lead to colonic motility, secretory, and absorptive dysfunction.

#### Autoimmune Denervation during *Trypanosoma*-Infection

The chronic autonomic nervous pathology observed in Chagas’ disease has an autoimmune basis. First, *T. cruzi* seems to induce loss of tolerance to self antigens in these tissues, and second, *T. cruzi* releases parasite antigens which cross-react with host antigens to lead to autoimmune responses (Pentreath, 1995). A variety of antibodies that cross-react with host tissues are found in the host upon *T. cruzi* infection, and antibodies against neurons seem particularly well represented (Pentreath, 1995). Indeed, among cross-reacting proteins, F1-160, a *T. cruzi* flagellar surface antigen that mimics intra-axonal filaments of myenteric nervous tissue has been described. Antibodies to this protein bind nervous tissues in both humans and rodents, and may cause lethal paralysis when transferred to non-infected mice (Petry and Van Voorhis, 1991). Moreover, patients with chronic Chagas disease present increased antibody responses to peripheral myelin components, such as myelin basic protein (MBP). MBP is recognized by T lymphocytes from patients with the digestive form of Chagas disease (Oliveira et al., 2009).

#### Neurotoxic Destruction of Neurons in Chagas Disease

*Trypanosoma*-induced neurolysis has been largely studied in animal models and the mechanisms remains unclear. In experimentally infected mice, there are areas in which the ganglionic neurons or the plexus are deformed or absent, while other areas exhibit normal anatomical features (Maifrino et al., 1999). Since neurons are not directly parasitized by *T. cruzi*, desctruction due to parasite invasion is unlikely (Köberle, 1968). Neurolysis can directly follow the rupture of pseudocysts, liberating disintegrating amastigotes, before the inflammatory reaction appears. It has been suggested that neuronal destruction could be due to a neurotoxin-like substance released by the disintegrating amastigotes (Köberle, 1968, 1970; Pentreath, 1995). Studies in *T. cruzi*-infected rats established a relationship between NO production and ganglion cell loss, as high NO synthase in the muscle layers of infected rats was associated with lower numbers of intramural neurons (Garcia et al., 1999). Production of IFN-γ and TNF-α in *T. cruzi* infected mice results in the activation of inducible iNOS and elevated NO synthesis, which is critical for trypanocidal activity in macrophages (Gazzinelli et al., 1992; Vespa et al., 1994). Infected IFN^−/−^ mice did not have significant neuronal loss, and present with no inflammatory infiltrate in the intestinal wall. In iNOS^−/−^ mice infected with *T. cruzi*, despite greater parasite accumulations and similar inflammatory infiltrates, numbers of myenteric plexus neurons remain unchanged, and neuronal nerve profile expression is preserved (Arantes et al., 2004). These observations suggest that intestinal denervation could either be secondary to the inflammatory processes or that NO could be a mediator of neuronal damage.

#### Immune Response-Dependent Neuronal Destruction and Enteric Glial Cell Destruction Contributes to Megacolon

The inflammatory infiltration in patients suffering from GI Chagas disease is comprised mainly of CD3-immunoreactive T lymphocytes and CD20-immunoreactive B lymphocytes (da Silveira et al., 2007). Natural killer cells (NK) and TIA-1 cytotoxic cells may also be found in colonic lesions of patients with megacolon, further asking whether the immune response may participate in neuronal degeneration.

Enteric glial cells are also decreased in the colon of patients with Chagas’ Disease (da Silveira et al., 2007, 2009) suggesting that neuronal destruction is associated with enteric glial cell destruction. Enteric glial cells play an active role in the control of gut physiology and pathophysiology, and participate actively in the course of intestinal inflammation (Aubé et al., 2006; von Boyen et al., 2004). More research is needed to assess the role of the loss of enteric glial cells in Chagas disease. Non dilated portions from chagasic patients with megacolon exhibited mild neuronal destruction and almost no signals of inflammation while dilated portions of the same organs showed more severe neuronal destruction because of a fulminating inflammatory response (da Silveira et al., 2009). In addition, enteric glial cells exposed to pro-inflammatory cytokines could control the inflammatory process *via* GFAP (von Boyen et al., 2004). Moreover, a recent study showed that enteroglial cells from chagasic patient with megacolon express HLA-DR complex class II, CD80 and CD86 components characteristic of antigen presenting cells (APCs; da Silveira et al., 2011). The authors speculate that enteroglial cells could play a pivotal role as facultative APCs and present native or foreign antigens to naive or effector CD4^+^ or CD8^+^ T lymphocytes (da Silveira et al., 2011). Clearly, a relationship between immunity and enteric glia does exist, and is crucial to the homeostatic maintenance of both systems. Findings to date also suggest that the inflammatory process and glial cell alterations observed in Chagas disease might disrupt the ENS and contribute to the development of pathology.

#### Neurotransmitter Implication in Megacolon

Neurons from the myenteric and submucosal plexuses of dilated colonic portions from chagasic patients present high level of SP and low levels of NK1R compared to non-dilated portions and to non-infected tissues which contribute to the maintenance of the inflammatory process in chagasic megacolon (Renzi et al., 2000; da Silveira et al., 2007, 2008b).

#### Consequences of Neuronal Destruction Induced by *Trypanosoma*

*Trypanosoma*-induced neuronal destruction appears to be selective as it affects primarily the medium sized and large neurons (Maifrino et al., 1999). Reduced acetylcholinesterase (AChE) activity has been recorded in the neurons and fibers of chagasic animals (Maifrino et al., 1999). *Trypanosoma*-induced denervation leads to hypersensitivity mediated at least in part by a decrease in AChE activity (Brennessel et al., 1985). This in turn decreases the responsiveness of the colonic smooth muscles to ACh, and consequently reduces the contractive capabilities of this muscle (Maifrino et al., 1999). The role of other enteric neurotransmitters in Chagas’ disease needs to be further assessed.

Intestinal peristalsis observed in patients with chagasic megacolon has also been associated with decreased IK channels (da Silveira et al., 2008a). IK channels are responsible for after-hyperpolarizing potentials in IPNs, thus altered expression of IK channels may result in altered timing of neural events in the reflex pathways.

In patients infected with *T. cruzi* and presenting with megaesophagus, similar processes are involved as dilatation implicates neuronal destruction in both plexuses, loss of enteroglial cells, and presence of inflammatory infiltrate containing NK and TIA-1 immunoreactive lymphocytes (da Silveira et al., 2005; Nascimento et al., 2010).

Finally, Interstitial cells of Cajal (ICCs) promote impulses of the ENS and neural inhibitory stimulation in different regions of the intestinal tract (Thuneberg, 1982). Recent studies have observed a significant reduction of ICCs in chagasic patients in both colon and esophagus (Hagger et al., 2000; Iantorno et al., 2007; de Lima et al., 2008). The role of these cells in the genesis of megacolon and megaesophagus warrants further investigation.

### Toxoplasma gondii

*Toxoplasma gondii* is an obligate, intracellular parasitic protozoan found worldwide. Although mild flu-like symptoms occasionally occur during the first few weeks following exposure, *T. gondii* infection does not usually produce observable symptoms in healthy humans. However infection can cause serious illnesses in immunocompromised hosts, infants, and pregnant women. Infection can also be transmitted transplacentally from the mother to the fetus. In the intermediate human host, *T. gondii* undergoes two phases of asexual developments: first tachyzoites multiply rapidly in many different types of host cells, and then initiate a second phase of development resulting in the formation of tissue cysts that have a high affinity for neural and muscular tissues. Cysts are common in the CNS, skeletal and cardiac muscle but can also be found in visceral organs (Tenter et al., 2000). Within the intestinal lumen, cysts release bradyzoites, and these parasitic forms interact with the intestinal epithelium and invade enterocytes, goblet cells and IELs.

Studies reported changes in the intestinal wall of infected animals, possibly due to the fact that the GI tract is the route of entry of *T. gondii*, and suggests that digestion and absorption may be compromised. Diarrhea has been reported in some models of toxoplasmosis (mice, dogs, pigs, sheep and goats; Odorizzi et al., 2010). Alterations in the myenteric plexus of *Toxoplasma*-infected animals have been described (Sugauara et al., 2008; Odorizzi et al., 2010; Hermes-Uliana et al., 2011; Silva et al., 2011; Papazian-Cabanas et al., 2012; Sant’Ana et al., 2012; Zaniolo et al., 2012; Araújo et al., 2015). In these studies, oral or intraperitoneal infection with *T. gondii* also disrupted myenteric neurons. Infection seems to affect the metabolism of the myenteric neurons probably suppressing the synthesis of cytoplasmic and nuclear proteins which could explain the smaller cell volumes of neurons observed in these studies (Sugauara et al., 2008; Silva et al., 2011).

#### *Toxoplasma gondii* Infection Induces Quantitative and Phenotypic Changes in Neurons

In swine models of toxoplasmosis, although the total number of nitrergic (NADPH-diaphorase-positive) neurons was not altered by infection, the number of neurons per ganglion increased (Odorizzi et al., 2010). It was suggested that part of these neurons, which at first did not produce NO (which colocalizes with NADPH-diaphorase), began to secrete it in response to the parasite-induced IFN-γ, the key cytokine for resistance against *T. gondii*-infection (Suzuki et al., 1988). The mechanisms of how IFN-γ alters the neurochemical expression of myenteric ganglia remain unclear. Infection with *T. gondii* also decreases the total number and the number of neurons *per* ganglion of the NADH-diaphorase-positive neurons (which are metabolically more active than the nitrergic neurons; Suzuki et al., 1988). As the NADH-diaphorase enzyme is mitochondria-bound, this would be consistent with a *Toxoplasma*-induced detrimental effect on myenteric neuron mitochondria. In the rat jejunum, toxoplasmosis reduces the number of goblet cells producing neutral mucins and sulphomucins, while maintaining sialomucin secreting cells, which results in the production of a more fluid mucus (Sant’Ana et al., 2012). The effect was concurrent with decreased numbers of VIP-immnunoreactive submucosal neurons. It was recently observed that rats infected *T. gondii* exhibit over 30% myenteric neuronal death, contain elevated proportions of nitrergic myenteric neurons, and no alteration of the NADHd-p neurons in the jejunum (Araújo et al., 2015). Increased variscosities with VIP nerve fibers were also seen in the myenteric plexus of infected animals (Araújo et al., 2015). As these VIP-fibers belong mainly to inhibitory motor neurons that produce NO, it remains to be seen whether this increase is related to an increase in NO production to recover homeostasis and intestinal motility in infected animals. Toxoplasmosis also causes enteroglial cell death, and these cells are known to play an important neurotrophic, anti-apoptotic and neuromuscular transmitter role. Enteric glial cell death may be a determining factor in the changes of the metabolic profile and chemical coding of surrounding neurons in toxoplasmosis. *T. gondii* was only detected in the mucosa and submucosa, suggesting that the alterations observed in the myenteric plexus are the result of an indirect action of the parasite possibly* via* host cytokines.

Taken together the observations in the myenteric neurons of *T. gondii*-infected animals indicate that the nervous system of different species show diverse responses to infection at different intestinal sites (duodenum, jejunum, ileum colon), different times (acute *vs*. chronic), and in response to different infective forms (tachyzoite, bradyzoite, sporozoite). However little is known of the mechanisms by which *T. gondii* induces myenteric neurons alterations. An effect of the host immune system, *via* IFN-γ or IL-12 production has been proposed in the control of multiplication and virulence of the parasite (Papazian-Cabanas et al., 2012). Further studies are needed to characterize how *Toxoplasma* affects the ENS, with particular emphasis on the implication of neurotransmitters such as SP, ACh, CGRP, VIP, the role of inflammatory mediators and enteroglial cells, and the consequences of their actions.

## Conclusion

In this review, we show that GI parasites can have important effects on gut functions by modifying the ENS. Table 1 summarizes the various mechanisms whereby enteric parasites may alter gastrointestinal neuroregulation. The two major alterations common to all the parasites presented here include altered intestinal contractility and altered ion and fluid transport. However the mechanisms involved remains incompletely elucidated and vary among species. Indeed some parasites will have a direct influence on the ENS by altering neuron numbers or phenotype; while others will influence neurotransmitter release that in turn modify the ENS activity (summary table). In addition a correlation between the host immune response, the inflammatory mediators and the ENS activity was shown for several parasites further underlying the importance of the immune-brain-gut axis. Beyond the need for mechanistic studies into these effects, directions for future research include the role of parasite-induced microbiota dysbiosis in gut neuroregulatory responses during and after infection.

**Table 1:** **This table provides an update on how enteric parasites may alter neuro-regulation pathway in the gut, and lists the physiological consequences that have been associated with these changes**.

Parasite	Physiological modifications	Neuroregulating factors involved	References
*Cryptosporidium parvum*	Altered ion transport	– Prostaglandins (PGE_2_)	Argenzio et al. (1993); Argenzio et al. (1996) and Laurent et al. (1998)
		– Prostacyclins (PGI_2_) via cholinergic and VIPergic nerves
	Malabsorption/Hypersecretion	– Increased levels of Substance P	Hernandez et al. (2007)
*Giardia duodenalis*	Altered intestinal contractility/motility	– Nitric Oxide depletion by *Giardia*^′^ arginine consumption	Barthó et al. (1992); Juanola et al. (1998); Eckmann et al. (2000); Leslie et al. (2003); Dizdar et al. (2010) and Pavanelli et al. (2010)
		– Reduced 5-HT
		– Increased CCK trigerred by mast cell
	Malabsorption/Hypersecretion	– Increased intestinal transit, reduction in villus and microvillus areas	Buret et al. (1992); Buret et al. (2015); Gorowara et al. (1992); Cevallos et al. (1995) and Troeger et al. (2007b)
	Hypersensitivity	– Correlation with *c-fos* activation and *Giardia*-induced bacterial translocation	Chen et al. (2013); Halliez et al. (2014); Balestra et al. (2012); Barbara et al. (2004) and Cenac et al. (2007)
		– Role for mast cells
*Entamoeba histolytica*	Neurons and axons degradation	– Cysteine-protease dependent degradation	Lourenssen et al. (2010)
*Nippostrongylus brasiliensis*	Motility dysfunctions	– Correlation with reduced *c-fos* expression	Crosthwaite et al. (1990); Goldhill et al. (1997); Castex et al. (1998); Urban et al. (1998); Gay et al. (2001); Gay et al. (2002); Akiho et al. (2002) and Zhao et al. (2003)
		– Inflammatory mediators influence
		– CCK action via CCK-A, CCK-B, cholinergic stimulation and vagal afferent pathway
		– IL-4Rα-activated Stat 6 dependent mechanism
	Nerve remodeling	– Mucosal nerves degeneration during the acute phase of inflammation in correlation with mast cell degranulation	Stead et al. (1991) and Stead (1992a)
		– Reinnervation after parasite expulsion
	Impaired fluid transport	– Probably linked to mast cell products action on ENS	Jodal et al. (1993)
	Chemo-/Mechanosensitivity	– Permeability changes/alteration in excitability of intrinsic neuronal reflexes	Aerssens et al. (2007); Holzer (2003); Schuligoi et al. (1998); Zhao et al. (2006) and McLean et al. (1997)
		– Sensitization of vagal afferent neurons
		– Alteration of genes profile of the vagal pathway involved in chemosensitivity
		– Decreased 5-HT_3A_ receptor
		– Correlation with mast cell hyperplasia
		– Dependent on tachykinin NK2 receptor
*Trichinella spiralis*	Altered intestinal contractility	– Muscle hypertrophy and hyperplasia	Vermillion et al. (1991); Blennerhassett et al. (1992); Hurst and Collins (1993); Kataeva et al. (1994); Rühl et al. (1994); Weisbrodt et al. (1994); Vallance et al. (1999); Khan and Collins (2006); Tanović et al. (2006); Keating et al. (2008) and Leng et al. (2010)
		– Mastocytosis
		– Altered neurotransmitter release (SP modulates GI inflammation, decreased NK1 immunoreactivity, pro-inflammatory cytokines production)
		– Altered 5-HT receptor function
		– role for T-lymphocytes
*Hymenolepis diminuta*	Altered intestinal contractility	– Integrity of the ENS necessary as sensors along the intestine initiate its activity	Dwinell et al. (2002)
	Altered ion transport	– Affected by histamine	McKay et al. (1991); Cooke (1994); McKay and Fairweather (1997) and Schultheiss et al. (2006)
		– Role for neurotransmitters (SP, CGRP)
*Schistosoma mansoni*	Altered intestinal contractility	– Direct effect via smooth muscle cell activation	Marquardt et al. (1984); Weinstock and Blum (1990a); Weinstock and Blum (1990b); Collins (1996); Fozard et al. (1996); Bogers et al. (2000); De Man et al. (2001); De Man et al. (2003); Moreels et al. (2001) and De Jonge et al. (2003b)
		– Indirect effect through activation of neuronal nicotinic receptors and alteration of myenteric plexus
		– Role for mast cells, pro-inflammatory cytokines and prostaglandins
		– Impairment of purinergic control of enteric cholinergic neurotransmission
		– SST receptor upregulation
	Increased intestinal muscle thickness	– Diffuse mucosal inflammation due to granulomas	Bogers et al. (2000) and Moreels et al. (2001)
		– Role for mast cells
*Trypanosoma cruzi*	Denervation/Decrease of enteric glial cells	– Loss of tolerance to self antigen	Köberle (1968); Pentreath (1995); Garcia et al. (1999); Lee et al. (1999) and da Silveira et al. (2007); da Silveira et al. (2009)
		– Cross reaction between parasite released antigens and host antigens (parasite antigens mimics hosts antigens)
		– Neurolysis due to neurotoxin-like substance released by disintegrating amastigote upon pseudocyst rupture
		– High NO levels and iNOS activation
		– Host immune response
	Hypersensitivity	– Decreased responsiveness of smooth muscle to ACh and consequently reduced contractive capabilities	Maifrino et al. (1999)
*Toxoplasma gondii*	Quantitative and phenotypic changes in neurons	– NO production by parasite-induced IFN-*γ*	Suzuki et al. (1988) and Araújo et al. (2015)
		– Parasite detrimental effect on mitochondria

## Funding

The authors are grateful for the funding support provided by the Natural Sciences and Engineering Research Council of Canada (NSERC), Discovery Grant #183681-2011, (www.nserc-crsng.gc.ca), and the Natural Sciences and Engineering Research Council of Canada Collaborative Research and Training Experience Program (NSERC CREATE), #413888-2012, www.nserc-crsng.gc.ca.

## Conflict of Interest Statement

The authors declare that the research was conducted in the absence of any commercial or financial relationships that could be construed as a potential conflict of interest.
